# 

**Published:** 1854-09

**Authors:** 


					﻿No. IV.]
SEP T EZlB E II.
[Vol. X.
BUFFALO
MEDICAL JOURNAL
AND
Jttuntljlt) lleuiciv
OF
MEDICAL AND SURGICAL SCIENCE.
AUSTIN FLINT, M. D., I
SANFORD B. HUNT, M. D., $ EDrr0BS;
BUFFALO, N. Y.
PUBLISHED BY JEWETT, THOMAS <k CO.
Commercial Advertiser Buildings.
1854.
’ce 82.50 per annum, in advance.
				

